# Microorganisms and Their Importance in the Food Industry: Safety, Quality and Health Properties

**DOI:** 10.3390/foods13101452

**Published:** 2024-05-08

**Authors:** Celia C. G. Silva, Susana Chaves Ribeiro

**Affiliations:** 1 Institute of Agricultural and Environmental Research and Technology (IITAA), University of the Azores, 9700-042 Angra do Heroísmo, Portugal; susana.ic.ribeiro@uac.pt; 2 School of Agrarian and Environmental Sciences, University of the Azores, 9700-042 Angra do Heroísmo, Portugal

Microorganisms can play an important role in food production, especially through fermentation processes [1]. Fermentation has been used not only as a strategy to preserve food but also to improve certain food properties, such as flavour, aroma, texture, and digestibility. Therefore, fermented foods have always been valued for their improved shelf life and unique flavour, aroma, texture, and safety [2,3]. In addition, traditional fermented foods can be a source of novel microorganisms with interesting properties that can be utilised by the food industry [4,5,6]. In recent decades, there has also been increasing scientific evidence that fermented foods and the microorganisms associated with them can positively affect human health [7,8,9,10,11,12]. Certain microorganisms benefit the host by correcting imbalances in the gut microbiota [13,14], stimulating immunity [15] and producing a variety of bioactive compounds including vitamins [16], enzymes [17], bacteriocins [18], bioactive peptides [19], conjugates of linoleic acid (CLAs) [20], short-chain fatty acids [21], gamma-aminobutyric acid (GABA) [22] and exopolysaccharides (EPSs) [23]. However, the safety of these microorganisms must be ensured as some of them may carry virulence factors that could be transferred to the commensal gut microbiota [24]. In addition, alternative food sources such as insects may contain a variety of new microorganisms whose safety must be ensured [25,26].

In this context, this Special Issue contains five research papers and two reviews dealing with the latest advances in the study of food microorganisms, from their potential contribution to improving the organoleptic properties and quality of food to their safety and use as probiotics to improve human health.

The review by Coelho et al. (contribution 1) examines the scientific literature on the role of lactic acid bacteria (LAB) in traditional raw milk cheeses in developing the specific characteristics of each type of cheese. The microbiota of raw milk cheeses is very complex and includes numerous strains of starter and non-starter lactic acid bacteria that are important for cheese ripening and flavour development. Protected Designation of Origin (PDO) cheeses, in particular, have a unique and more intense flavour compared to pasteurised milk cheeses, which has sparked interest in studying the structural and functional diversity of the microbial ecology of these cheeses. Identifying the microorganisms established in PDO cheeses is crucial to studying the contribution of the production conditions to the formation of the microbial communities and exploring the influence of the metabolically active microbiota on the development of the sensory characteristics of the cheese. This review also describes the potential applications of LAB and their metabolites in food preservation, as some strains produce antimicrobial metabolites that prevent the development of pathogenic and spoilage bacteria, moulds, and yeasts. In addition, the health benefits of certain LAB strains as producers of bioactive compounds and recent advances in using LAB to promote human health are described.

In the work presented by Rocha et al. (contribution 2), the microbiota of an artisanal PDO cheese—Serra da Estrela PDO—is analysed using recent methods. This Portuguese cheese is made from raw sheep’s milk using traditional methods in small dairies and is highly appreciated by consumers. The first step in protecting the microbial biodiversity of traditional PDO cheeses is to know it. In this study, the authors present an in-depth assessment of the LAB communities of PDO Serra da Estrela cheeses and raw materials (raw sheep’s milk and use of cardoon as a vegetable coagulant). The evaluation of the microbiological indicators of hygiene and food safety showed that Serra da Estrela cheese is a safe food. This study also identified a group of five species, *Lacticaseibacillus paracasei*, *Leuconostoc mesenteroides*, *Lactococcus lactis*, *Enterococcus durans*, and *Enterococcus faecium*, which are essential for the production of PDO Serra da Estrela cheese. This study also contributed to isolating an autochthonous set of starters and adjunct cultures to produce Serra da Estrela cheese.

Food can be contaminated with pathogenic microorganisms on its way through the supply chain and thus endanger consumers’ health. Therefore, safe food handling practises, and procedures must be implemented at all stages of food production to minimise these risks and prevent foodborne illnesses. In the work presented by Iacumin et al. (contribution 3), the microbial contamination of cocoa powder and chocolate bars sold in Italy was analysed. They found that the spore-forming microorganisms isolated from cocoa and chocolate bars probably originate from the raw material, as they can survive the extraction and manufacturing processes in the cocoa supply chain. In contrast, the origin of the moulds found in chocolate bars was found to be related to the production environment, occurring in the final stages of production and packaging. This study could be useful for the food industry to better evaluate the preventive measures in food establishments and improve the official control phases related to food safety.

In the search for alternative protein sources, insects have become the focus of scientific research [27]. Several studies have already been conducted on the yellow mealworm—*Tenebrio molitor*—as a safe alternative protein source for human nutrition [28,29]. However, there is a need to assess the microbial safety of these insects and insect products sold for human consumption. Therefore, the microbiological contamination of mealworms is the focus of the study by Pöllinger-Zierler et al. (contribution 4). The authors analysed a possible link between the microbiological contamination of mealworms and the substrates, as well as the best processing methods to ensure the risk-free consumption of these insects. They concluded that the choice of substrate does not influence the microbial contamination of mealworm larvae. Furthermore, the evaluation of different processing steps to reduce the overall microbiological load showed that heating and starvation allow the risk-free consumption of these insects.

Indigenous bacteria isolated from food can be a safe source of enzymes that can be used in the food industry to improve flavour and texture. In this context, Wang et al. (contribution 5) identified new bacteria isolated from fish that can synthesise esters. One bacterial strain, *Acinetobacter venetianus* SCSMX-3, was isolated from fermented golden pomfret and showed high ester synthase activity with good growth under mesophilic conditions. Genome sequencing and gene function analysis indicated a considerable number of genes related to energy metabolism and flavour synthesis. In addition, enzymes involved in lipid metabolism were identified in this strain, including a triacylglycerol lipase belonging to the abH15 lipase superfamily. This study expands the microbial sources of carboxylate hydrolases, as these new strains can be used to improve flavour in fermented fish products.

In addition to technological applications, microorganisms isolated from food can also be a valuable source of bioactive compounds. In Ribeiro et al.’s (contribution 6) study, LAB strains isolated from *Chlorella vulgaris* photobioreactors were analysed to produce vitamin B12 (cobalamin biosynthesis) and probiotic potential. Of the three bacterial strains identified as potential cobalamin producers, *Pediococcus pentosaceus* L51 was the highest producer and a promising candidate for developing high vitamin B12 formulations.

Consumers are becoming increasingly aware of the positive effects of microorganisms on human and animal health, which has led to an increasing demand for probiotic products worldwide. The review by Kieps and Dembczynski (contribution 7) describes the current trends in producing probiotic formulations to be consumed by animals and humans. This review discusses the different drying methods and their improvements, with particular interest in the process conditions, microorganisms, and protective substances. In addition, the factors (thermal, osmotic, oxidative, and acidic stress, dehydration, and shear forces) that influence the quality and stability of the final probiotic preparations are discussed, and some alternatives to mitigate the effects of these factors are presented.

To summarise, microorganisms in food can play a very important role, from food spoilage, poisoning, and infection to food preservation and production. In addition, microorganisms in food may provide health benefits and can be consumed as probiotics. Research on probiotics has made considerable progress in recent years, driven by the understanding of the effects of the gut microbiota on human health [30]. However, further human studies are needed to provide convincing evidence of the benefits of probiotics for human health.
